# Bu Shen Huo Xue decoction restores endometrial leukemia-inhibitory factor but not Angiopoietin-2 expression, and improves uterine receptivity in the controlled ovarian stimulation rat model

**DOI:** 10.3892/etm.2015.2193

**Published:** 2015-01-20

**Authors:** XIN GONG, JIAOYING LOU, QIUDAN LU, HAITAO HUANG, ZHE JIN

**Affiliations:** Reproductive Endocrinology Center, Dongfang Hospital, Beijing University of Chinese Medicine, Beijing 100078, P.R. China

**Keywords:** chinese herbs, angiogenesis, implantation, leukemia-inhibitory factor, angiopoietin-2

## Abstract

Leukemia-inhibitory factor (LIF) and Angiopoietin-2 (Ang-2) are important factors in fertility. In the present study, it was investigated whether Bu Shen Huo Xue Decoction (BSHXD) prevents controlled ovarian hyperstimulation (COH) treatment-induced changes in endometrial LIF and Ang-2 expression and whether it has an effect on the number of implantation sites and live births in rats. Uteri were collected on day (D) 3, 4 and 5 of pregnancy, and LIF and Ang-2 protein and mRNA expression were detected using western blot analysis and quantitative polymerase chain reaction. On pregnancy D10, the average number of implantation sites was observed. The number of live births from each group was recorded. The results indicated that BSHXD treatment markedly increased the number of live births by restoring endometrial LIF expression and the implantation capacity in the COH rat model. In addition, no association was identified between LIF and Ang-2 expression. Therefore, this suggests that BSHXD may be useful for female reproduction.

## Introduction

Implantation is a critical step during normal pregnancy. The implantation process occurs after 4–6 days of pregnancy in rats (plug presented day = gestation day 1) (1), and a similar phenomenon also occurs in humans between days luteinizing hormone (LH)+6 and LH+8 (2). Successful implantation requires a receptive endometrium, a normal embryo at the blastocyst developmental stage and a synchronized dialogue between maternal and embryonic tissues (3).

A prerequisite for implantation is increased endometrial angiogenesis and vascular remodeling at the implantation site. Leukemia-inhibitory factor (LIF) is a well-characterized cellular factor that is a promising candidate as an endometrial receptivity biomarker in mice (4) and humans (5,6). LIF knock-out female mice are infertile, and implantation of the embryo does not occur (7). LIF has a role in the adhesive and invasive phases of implantation (8). Angiopoietin-2 (Ang-2) destabilizes the quiescent endothelium and primes it to respond to exogenous stimuli, thereby modulating angiogenic cytokine activity (9,10). Ang-2 is expressed in the peri-implantation endometrium in a spatiotemporal manner and participates in angiogenesis and the vascular remodeling process (11). Although LIF and Ang-2 are essential for normal blastocyst implantation, it is not yet clear whether there is an association between LIF and Ang-2.

The controlled ovarian hyperstimulation (COH) with gonadotropin-releasing hormone agonist (GnRHa) long protocol is an important approach in IVF. COH results in high-quality embryos; however, even with ongoing advances, implantation rates are still relatively low (12). A number of studies have demonstrated that COH may directly change endometrial characteristics compared with those of the natural cycle (13–15), and these differences may alter endometrial receptivity (16). Furthermore, high serum estradiol levels or other hormonal alterations that result from COH may indirectly adversely affect implantation (17,18).

Although the current understanding of implantation has increased, therapeutic options remain limited (19). Further study is required to investigate clinical treatment options for infertility patients with implantation failure. In China, traditional Chinese medicine (TCM) harmonizes the endocrine environment to assist with assisted reproductive technology (20–22), and integrating the principles and knowledge from TCM may be useful in clinical practice to provide better strategies for treating infertility (23). Therefore, in the present study, Bu Shen Huo Xue Decoction (BSHXD) was established to aid in preparing the endometrium for implantation (Table I).

In the present study, endometrial LIF and Ang-2 protein and mRNA expression was investigated, and the number of implantation sites and live births in a COH rat model were determined to elucidate the side-effects of COH on fertility. It was hypothesized that BSHXD can ameliorate these side-effects and improve endometrial receptivity and pregnancy outcome. The results may therefore provide evidence to support the use of BSHXD in assisted reproduction.

## Materials and methods

### Ethics statement

All the experimental protocols were approved by the Ethics Committee of the Beijing University of Chinese Medicine Animal Care and Use Committee (no. 2012-087-R; Beijing, China).

### BSHXD preparation

The drugs present in BSHXD were obtained from the Pharmacy Department of Dongfang Hospital of Beijing University of Chinese Medicine (Beijing, China). The quality of the raw herbs was controlled according to the requirements of the Pharmacopoeia of the People’s Republic of China. An aqueous extract of BSHXD was prepared in accordance with the following procedure. In brief, the components (as shown in Table I) were mixed in proportion and were macerated for 1 h with 8 volumes of distilled water and then decocted for 2 h. The cooled extract was filtered. The extraction procedure was repeated twice. The extracts were then combined and concentrated by boiling to a final volume of 100 ml (4.12 g/ml). This dilution was used in the following preliminary experiments in a range of concentrations (between 1.03 and 4.12 g/ml).

### Treatment

Female Sprague Dawley rat virgins aged 7–8 weeks old (weighing 210–220 g) were maintained in the laboratory on a 12 h light, 12 h dark regimen with free access to water and a standard diet. The estrous stage was identified by vaginal smear. Only female rats with regular cycles were used. The rats were randomly allocated into four groups: control, COH, BSHXD and COH+BSHXD groups (n=30 in each group). A total of 18 rats from each group were used for the western blot and quantitative polymerase chain reaction (qPCR) analyses, 6 rats were used to assess the implantation site number and 6 rats were used to assess pregnancy outcomes.

Rats in the COH group were administered 1 ml/100 g body weight/day distilled water for 12 days and treated with the GnRHa long protocol. In brief, a GnRH agonist (Diphereline; Ipsen Pharma Biotech, Signes, France) was injected intraperitoneally at 1.5 μg/100 g body weight/day between the third and ninth day of estrous. Pregnant mare’s serum gonadotropin (Chifeng Bo’En Pharmaceutical Co. Ltd., Chifeng, China) was injected intraperitoneally at 5 IU/100 g body weight between the third and ninth day of estrous followed by 10 IU/100 g human chorionic gonadotropin (hCG; Yantai North China Pharmaceutical Co., Ltd., Yantai, China) after 28 h. In the BSHXD group, the animals were administered 1 ml BSHXD/100 g body weight/day for 12 days followed by saline injections at the same time and volume as the COH group. Animals in the COH+BSHXD group were administered 1 ml BSHXD/100 g body weight/day for 12 days and were then subjected to the same GnRHa long protocol as the COH group. In the control group, the rats were administered 1 ml distilled water/100 g body weight/day for 12 days, followed by saline injections at the same time and volume as the COH group. The female rats were housed overnight with males (1:1) following hCG or saline administration. Successful mating was assessed daily by the presence of a vaginal plug. The day that the plug was first observed was designated as day 0 of gestation (D0).

On each of D3, D4 and D5, 6 rats were sacrificed from each group. The uteri were removed without excess fat and connective tissue and the whole sample was stored at −80°C until protein and mRNA extraction.

### Western blot analysis

The uterus was sectioned, and slices were incubated and lysed in RIPA lysis buffer (C1053; Applygen Technologies, Beijing, China) supplemented with protease inhibitor (P1265; Applygen Technologies). The protein concentration was quantified with bicinchoninic acid (P1511; Applygen Technologies). Sodium dodecyl sulfate-polyacrylamide gel electrophoresis was performed using a 10% polyacrylamide gel, and the samples were transferred to nitrocellulose membranes (Bio-Rad, Hercules, CA, USA). Membranes were blotted with anti-LIF (sc-1336; Santa Cruz Biotechnology, Heidelberg, Germany) or anti-Ang-2 (AP23297PU-N; Acris Antibodies, Herford, Germany) primary antibodies at a 1:1,000 dilution and incubated overnight at 4°C. Following incubation, the membranes were washed 3 times with Tris-buffered saline and Tween 20 buffer and then incubated with the secondary antibodies (P1308, P1309; Applygen Technologies) at a 1:10,000 dilution at room temperature for 1 h. The blots were visualized using the Super ECL Plus detection reagent (P1010; Applygen Technologies). The enhanced chemiluminescence signals were detected using Quantity One software (Bio-Rad). GAPDH (blotted with ab8245; Abcam, Cambridge, UK) was used as an internal control to validate the quantity of protein loaded onto the gel.

### qPCR analysis

LIF and Ang-2 gene expression was measured using qPCR. Total RNA was extracted from the uteri of the control, COH, BSHXD and COH+BSHXD rats using TRIzol (Invitrogen Life Technologies, Carlsbad, CA, USA), in accordance with the manufacturer’s instructions. The RNA was thawed on ice and quantified spectrophotometrically; the quality was assessed using sodium dodecyl sulfate-agarose gel electrophoresis. Reverse transcription was performed with 8 μl total RNA per 20 μl reaction using a standard cDNA synthesis kit (Takara Bio, Otsu, Japan). Target gene primer sequences are listed in Table II.

For each qPCR assay, the thermal cycling conditions included an initial activation step at 95°C for 5 min, 40 amplification cycles and a final melting curve (65–95°C). PCR reactions were performed on an ABI Prism 7700 Sequence Detection System (Applied Biosystems, Foster City, CA, USA). Target mRNA levels were normalized against those of GAPDH. Target mRNA expression was analyzed using the 2^−ΔΔCt^ algorithm.

### Implantation sites and live births

On D10, 6 rats from each group were sacrificed. The uteri were removed without excess fat and connective tissue, and the conceptuses were removed from the uteri. The number of implantation sites in the uterine horn was recorded. The average number of implantation sites was calculated as the total number of implantation sites/number of rats. Following conception and birth, the number of newborn rats from each group was recorded. The average number of live births was calculated as the total number of newborn rats/number of rats.

### Statistical analysis

The data are presented as the mean ± standard error of the mean. One-way analysis of variance and least significant difference tests were used. P<0.05 was considered to indicate a statistically significant difference. Graphs of the data were produced using Microsoft Excel software.

## Results

### Western blot analysis

**Figure 1:**
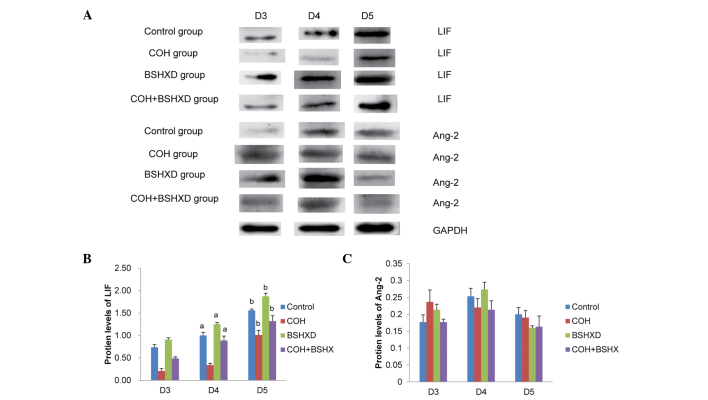
(A) Western blot analysis of LIF and Ang-2 protein expression in the four groups. (B) LIF and (C) Ang-2 protein expression levels were quantified using densitometry. All of the densities were normalized against GAPDH. ^a^P<0.05, compared with LIF protein expression on D3; ^b^P<0.05, compared with LIF protein expression on D4. Data are expressed as the mean ± standard error of the mean (n=6 in each group). LIF, leukemia-inhibitory factor; Ang-2, angiopoietin-2; COH, controlled ovarian hyperstimulation; BSHXD, Bu Shen Huo Xue Decoction; D, day.

**Figure 2:**
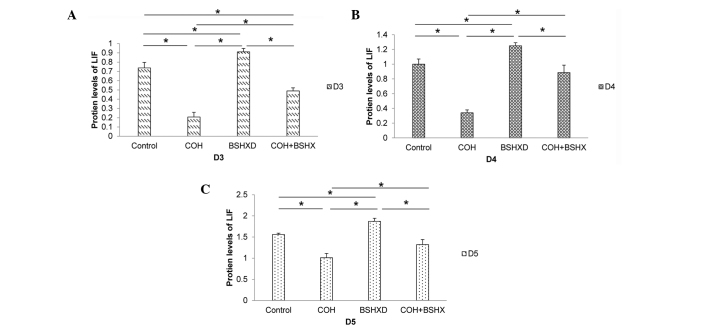
LIF protein expression levels in the four groups on (A) D3, (B) D4 and (C) D5. ^*^P<0.05. Data are expressed as the mean ± standard error of the mean (n=6 in each group). LIF, leukemia-inhibitory factor; COH, controlled ovarian hyperstimulation; BSHXD, Bu Shen Huo Xue Decoction; D, day.

Endometrial LIF and Ang-2 protein expression levels during implantation were determined using western blot analysis. The LIF and Ang-2 protein expression levels were normalized against those of GAPDH (Fig. 1). The LIF protein expression levels were found to be increased during the implantation period in the four groups (Fig. 2). However, COH treatment significantly reduced the level of LIF protein expression in the COH group compared with that in the other groups. No significant difference was identified between the LIF protein levels in the control and the COH+BSHXD groups on D4 and D5. Compared with the control and the COH+BSHXD treatment, BSHXD treatment significantly increased the level of LIF protein expression in the BSHXD group. However, no significant differences in the level of Ang-2 protein expression were observed from D3 to D5.

### qPCR

**Figure 3:**
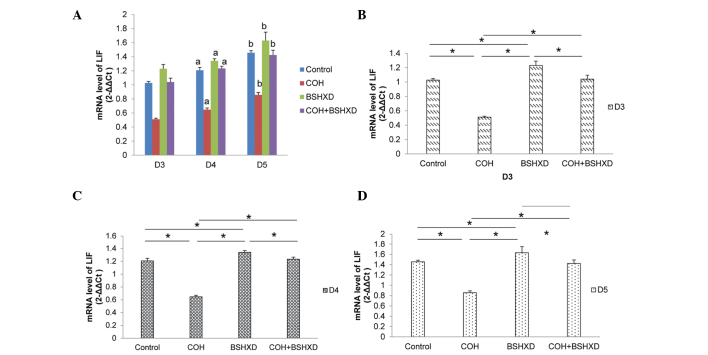
(A) LIF mRNA analysis by quantitative polymerase chain reaction during the implantation window in rats. ^a^P<0.05, compared with LIF mRNA expression on D3; ^b^P<0.05, compared with LIF mRNA expression on D4. LIF mRNA expression in the four groups on (B) D3, (C) D4 and (D) D5. ^*^P<0.05. Data are expressed as the mean ± standard error of the mean (n=6 in each group). LIF, leukemia-inhibitory factor; COH, controlled ovarian hyperstimulation; BSHXD, Bu Shen Huo Xue Decoction; D, day.

**Figure 4:**
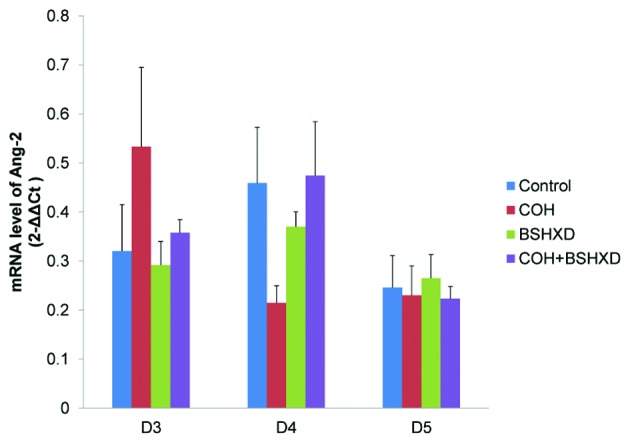
Ang-2 mRNA analysis by quantitative polymerase chain reaction during the implantation window in rats. Data are expressed as mean ± standard error of the mean (n=6 per group). Ang-2, angiopoietin-2; COH, controlled ovarian hyperstimulation; BSHXD, Bu Shen Huo Xue Decoction; D, day.

To verify the changes in expression levels, LIF and Ang-2 transcript levels were measured using qPCR. The differences in the qPCR results were statistically significant, and substantial differences in expression were observed using western blot analysis. LIF and Ang-2 mRNA were expressed in the rat endometrium during implantation. LIF mRNA expression levels were increased from D3 to D5 (Fig. 3). COH treatment significantly reduced the LIF mRNA expression levels compared with those in the control, BSHXD and COH+BSHXD groups, and the BSHXD group had significantly increased LIF mRNA expression levels compared with the control and COH+BSHXD groups. However, no significant differences were identified in Ang-2 mRNA expression levels from D3 to D5 (Fig. 4).

### Number of implantation sites and live births

The effects of COH and BSHXD on the number of implantation sites and live births are summarized in Table III. The number of implantation sites and live births in the COH group was significantly lower compared with those in the other groups. No significant difference was identified among the control, BSHXD and COH+BSHXD groups; however, the implantation site and live-birth number was highest in the BSHXD group.

## Discussion

The aim of the present study was to investigated the effect of BSHXD in a COH rat model during the implantation window using western blot and qPCR analyses and by measuring the average number of implantation sites and live births.

In numerous mammalian species, including mice, humans and sheep, uterine LIF expression is upregulated during the onset of embryo implantation (24), suggesting that LIF may be of general significance to implantation in mammals. Previous studies on early murine pregnancy have demonstrated that LIF expression levels are highest in the uterus between the fourth and fifth day of pregnancy (25). These data are consistent with the results from the present study that demonstrated that LIF expression increased during implantation in the four groups and peaked on D5. A role for LIF in implantation regulation and embryonic development was proposed. The observation that recombinant LIF inhibits murine embryonic stem cell differentiation (26,27) indicates that LIF may have a role in early embryonic growth and development. LIF mRNA is expressed in murine embryos from the fertilized egg to the blastocyst stage (28,29). These data likely explain why lower LIF protein and mRNA expression in the COH group was associated with lower implantation site numbers. Furthermore, LIF may also be important during placentation and subsequent fetal development. A potential role for LIF in placentation was first suggested when the LIF receptor (LIFR) was cloned from a human placental cDNA library (30), which was further strengthened by a study demonstrating that normal placentation was disrupted in LIFR^−/−^ mouse embryos (31). A possible explanation for the lower numbers of live births in the COH group in the present study is that the establishment of decidualization and placentation requires LIF; however, COH treatment significantly disturbed LIF expression. This indicates that LIF may have an important role in pregnancy establishment.

The other major finding of the present study was that there were no significant differences in Ang-2 expression among the four groups. Angiopoietins are a family of growth factors that promote vessel maturation and remodeling (32,33), which are important processes during implantation (34–36). Ang-2 causes loosening of cell-matrix and cell-to-cell contacts, which allows access to angiogenic inducers (37). Therefore, Ang-2 expression may promote angiogenesis (38). Furthermore, Ang-2 is selectively expressed in the ovary, uterus and placenta of mice and humans (32). Thus, it was hypothesized in the present study that Ang-2 expression would demonstrate a similar increase to LIF during implantation. However, this association was not observed. The lack of a difference in endometrial Ang-2 expression from D3 to D5 is that angiogenic mechanisms during implantation may not require Ang-2. In a previous study, Ang-2 expression levels were determined using *in situ* hybridization and it was demonstrated that Ang-2 was expressed at low levels between the first and fifth days of pregnancy (peri-implantation) and was then expressed at higher levels from the sixth to seventh day in mice (11). These results clearly demonstrate that Ang-2 may not be a dominant angiogenic factor during the early stages of pregnancy. The results of the present study support the theory that maternal regulation of endometrial angiogenesis prior to implantation is different from the regulation that occurs once the embryo has implanted (39).

It has been hypothesized that using COH with GnRHa in a long protocol to induce multifollicular development may also affect endometrial receptivity (40,41). The endometrium undergoes a morphological advancement prior to implantation, and it is not surprising that a side-effect of COH is the regulation of supraphysiological levels of steroid hormones and paracrine mediators produced and received by the endometrium. Genomic analyses of human endometrial receptivity have been previously performed (42,43), and these analyses demonstrate that numerous genes are aberrantly expressed in the COH endometrium, and the expression levels are similar to those in a non-receptive endometrium (44). These previous studies highlight the necessity for modifying COH treatment to achieve an endometrium that resembles that of the natural endometrium cycle morphologically and functionally, which is likely to improve pregnancy outcomes. Therefore, BSHXD was used along with COH treatment to promote the recovery of the impaired endometrium in rats. In a previous study, the authors of the present study demonstrated the effects of BSHXD on endometrial morphology and LIF expression in non-pregnant rats (45). The results from the present study, including increased LIF expression, and restored implantation site and live-birth numbers in the COH+BSHXD group, suggest that BSHXD treatment may be a clinical option for patients with an impaired endometrium following COH treatment. TCM uses a holistic and synergistic approach to restore homeostasis (46); however, TCM does not translate the normal state into a super-normal one, which accounts for a lack of significant differences between the control and BSHXD groups.

In conclusion, it was demonstrated that: i) COH treatment significantly decreased LIF protein and mRNA expression levels and the number of implantation sites and live births in rats compared with those in the natural cycle; ii) integrating BSHXD and COH treatment restores LIF expression and significantly increases the probability of implantation and the number of live births; iii) no significant differences in LIF expression or the average number of implantation sites and live births were observed among the control, BSHXD and COH+BSHXD groups; iv) LIF and Ang-2 were expressed in mature female rat endometrium during the implantation window; however, no association was found between LIF and Ang-2 expression. The results from the present study provide novel insights into a TCM approach for infertility treatment and assisted reproductive technology. However, further clinical studies are required to confirm the proposed approach.

## Figures and Tables

**Table I:** Bu Shen Huo Xue Decoction (BSHXD) composition.

Component		Ratio
(1)	Sheng Di [*Rehmannia glutinosa* (Gaertn.) Libosch., root]	15
(2)	Dan Shen (*Salviae miltiorrhizae* Bge., root)	10
(3)	Dang Gui [*Angelica sinensis* (Oliv.) Diels., root]	12
(4)	Chuan Duan (*Dipsacus asperoides* C. Y. Cheng et T .M. Ai., root)	15
(5)	Du Zhong (*Eucommia ulmoides* Oliv., cortex)	12
(6)	Shan Yao (*Dioscorea opposita* Thunb., rhizome)	15
(7)	Mei Gui-hua (*Rosa rugosa* Thunb., flower)	6
(8)	Chuan Xiong (*Ligusticum chuanxiong* Hort., rhizome)	6
(9)	Yi Yi-ren [*Coix lacryma-jobi* L. var. ma-yuen (Roman.) Stapf., seed]	12

**Table II:** Quantitative polymerase chain reaction primer sequences.

Gene	Primer sequence 5′→3′	Length	Amplicon
LIF	F: CCCTTCCCATCACCCCTGTA	20	102 bp
	R: TGCCGTTGAGTTGAGCCAGT	20	
Ang-2	F: CGGACTCTGTCACAAGCAAGAA	22	237 bp
	R: AGCACAAGACGGAACAACGAA	21	
GAPDH	F: TGCTGAGTATGTCGTGGAG	19	288 bp
	R: GTCTTCTGAGTGGCAGTGAT	20	

LIF, leukemia-inhibitory factor; Ang-2, angiopoietin-2; F, forward; R, reverse.

**Table III:** Number of implantation sites and live births in each group.

Variables	Control	COH	BSHXD	COH+BSHXD
Implantation sites	8.67±1.93	3.60±0.51^a^	9.14±1.18	7.83±0.48
Live births	9.00±1.90	3.17±0.40^a^	9.50±0.56	7.33±0.49

COH, GnRHa long protocol-stimulated rats; BSHXD, rats that received BSHXD treatment; COH+BSHXD, GnRHa long protocol-stimulated rats that received BSHXD treatment. Values are expressed as the mean ± standard error of the mean (n=6 in each group).

a P<0.05, versus all the groups.

COH, controlled ovarian hyperstimulation; GnRHa, gonadotropin-releasing hormone agonist; BSHXD, Bu Shen Huo Xue Decoction.
